# Regulating genome language models: navigating policy challenges at the intersection of AI and genetics

**DOI:** 10.1007/s00439-025-02768-4

**Published:** 2025-09-16

**Authors:** Bahrad A. Sokhansanj, Gail L. Rosen

**Affiliations:** 1 Department of Electrical and Computer Engineering, College of Engineering, Drexel University, Philadelphia, PA 19104 USA; 2 Law Office of Bahrad Sokhansanj, Los Angeles, CA 90034 USA

## Abstract

Genome Language Models (GLMs) represent a transformative convergence of artificial intelligence (AI) and genomics, offering unprecedented capabilities for biological discovery, healthcare innovation, and therapeutic design applications. However, these powerful tools create novel regulatory challenges that existing frameworks—whether AI governance or genomic privacy protections—cannot adequately address alone. This paper examines the critical regulatory gaps emerging at this intersection, highlighting tensions between AI principles that favor broad data access and genomic governance that demands stringent privacy protections and informed consent. We analyze how GLMs challenge conventional regulatory approaches as they pertain to applications in disease risk prediction, international research collaboration, and open-source model distribution. We propose a multilayered governance framework that combines policy innovations such as regulatory sandboxes and certification frameworks with technical solutions for privacy preservation and model interpretability. By developing adaptive governance strategies that bridge AI and genomic regulation, we can enable responsible GLM innovation while safeguarding individual rights, promoting equity, and addressing emerging biosecurity concerns in this rapidly evolving field.

## Introduction

Our 3.2 billion base pairs—and the billions more in other living organisms—contain the fundamental instructions for life. For decades, scientists have worked to decipher these complex codes, gradually uncovering powerful insight into evolution, biology, and disease (Lander 2011). Now, a revolutionary convergence of genomics and large language models (LLMs), has produced Genome Language Models (GLMs) (Benegas et al. 2023; Boshar et al. 2024; Consens et al. 2025). GLMs promise to transform our understanding of biology and revolutionize healthcare. Yet, as these powerful AI tools emerge, they create novel regulatory challenges that existing policy frameworks can struggle to address.

GLMs represent a paradigm shift in genomic analysis. Traditional methods identify specific patterns through sequence alignment/similarity-based analysis techniques and motif discovery (Bailey et al. 2009; Altschul et al. 1990; Steinegger and Söding 2017; Eddy 1998). These approaches excel at finding well-characterized elements but often miss complex interactions. GLMs instead treat the genome as a form of biological “text” with its own syntax and semantics. They use the same neural architecture that powers modern AI systems like ChatGPT to learn patterns directly from vast genomic datasets without requiring explicit annotation.

The power of GLMs is that they are *foundation* models: large, general-purpose models trained on broad datasets that can be adapted to a wide range of downstream genomic tasks (Moor et al. 2023). GLMs can capture both local and long-range interactions within genomic sequences, enabling unprecedented capabilities in variant effect prediction, regulatory element identification, and even synthetic sequence generation (Consens et al. 2025). GLMs thus exist at a critical and potentially problematic intersection between AI systems and genomic research, as they can contain and generate both personal and group-specific genetic information (Thomas et al. 2024).

This paper examines the regulatory gaps that emerge when neither AI governance principles nor genomic privacy protections can fully address the unique characteristics of GLMs. Critical tensions exist—from conflicts between broad data use and informed consent to challenges in model interpretability and cross-border collaboration. The paper proceeds as follows: First, we present illustrative scenarios drawn from our research group’s experience developing GLMs to show how they can trigger real-world regulatory conflicts and motivate the need to adapt governance frameworks. Second, we provide technical background on GLM architecture and capabilities. Third, we review the regulatory landscapes in both AI and genomics. Fourth, we analyze how GLMs fall into the gaps between regulatory regimes. Finally, we propose a blended governance approach that combines policy innovations with technical solutions to support the responsible development of this emerging technology.

## Illustrative scenarios of regulatory issues raised by the genome language model (GLM)

The regulatory gaps created by GLMs become particularly evident when examining concrete scenarios where AI governance and genomic governance collide. Table 1 summarizes the key regulatory tensions that emerge in these scenarios, highlighting how traditional AI and genomic viewpoints conflict when applied to GLMs. The following three scenarios illustrate how the tensions between these frameworks manifest in practice, motivating the development of targeted solutions that bridge these disparate regulatory domains. For the purposes of these scenarios, issues have been simplified; detailed discussions and citations will follow in later sections.

**Table 1:** regulatory tensions in GLM applications: summary of the GenePredictAI scenarios

Scenario	AI viewpoint	Genomics viewpoint	Regulatory conflict
1.a) Disease risk inferred from training data	No requirement to return incidental findings	Consent may not cover feedback	Duty to return results unclear
1.b) Upload reveals genetic similarity to others	User choice = user responsibility	Genetic privacy extends to relatives	Consent and privacy boundaries breached
2. International development across jurisdictions	Focus on algorithm review and export	Data export tightly controlled	Data can't move, model can't train across populations
3. Open-source model release	Promotes transparency and collaboration	Risks re-identification or misuse	Public model weights may leak sensitive genomic info

The fictional GenePredictAI system discussed in the accompanying text illustrates how GLMs challenge both AI and genomic governance frameworks. Each scenario reveals potential mismatches in expectations, predictions, and regulatory responsibility, highlighting the need for an integrative approach to oversight

### Scenario 1 disease risk prediction and clinical decision-making

Imagine “GenePredictAI,” a GLM created to analyze whole genome sequences and flag potential disease risks. The company developing this tool trains it on thousands of genomes from a biobank, where participants originally consented to have their data used for “health research.”

From an AI governance perspective, GenePredictAI would likely be classified as a high-risk system under frameworks like the EU AI Act, triggering requirements for accuracy testing, algorithmic impact assessments, and transparency about how predictions are made. Current AI regulations would focus on whether the model technically performs as advertised and whether its decision-making can be explained. By contrast, from a genomic governance standpoint, regulators would apply privacy laws like the HIPAA in the United States or GDPR in the European Union, examining whether the original consent adequately covers this specific application and if privacy safeguards meet the heightened standards required for genetic information. They would prioritize data security, access controls, and consent management.

The regulatory gap becomes evident in scenarios where neither framework provides adequate guidance, as in the following two examples.

(A) GenePredictAI unexpectedly identifies a strong correlation between certain genetic patterns and early-onset Alzheimer's disease—a finding not previously known to science. The current approach to AI regulations would typically permit such novel discoveries as beneficial innovation, while genomic frameworks would remain unclear on the duty to return incidental findings from research datasets.

*Proposed solution*: A regulatory sandbox approach, as proposed in this paper could provides a controlled environment to develop appropriate protocols for handling such discoveries while balancing innovation with ethical obligations.

(B) A customer uploads their genome to GenePredictAI for analysis, the system flags genetic similarities to people in the training data, potentially revealing biological relationships neither party consented to discover. Presently, neither AI nor genomic regulations adequately answers the question of who bears responsibility for this privacy breach: the model developer, the healthcare provider using the system, or the institution that supplied the training data?

*Proposed solution*: A certification framework could establish clear liability allocation and privacy standards specific to GLMs. This includes restricting how GLMs are used to prevent outputs that infer or reveal nonconsensual information.

### Scenario 2 cross-border research collaboration with conflicting regulations

GenePredictAI is being developed by a consortium bringing together researchers from the EU, U.S., China, and India. They face conflicting regulatory requirements (simplified here for illustrative purposes):

- European partners must comply with GDPR, which requires specific consent for each use of genetic data and places strict limits on data transfer outside the EU.
- U.S. researchers work under HIPAA and institutional review board requirements, which allow broader data use with appropriate anonymization.
- Chinese team members face new AI regulations requiring government review of algorithms and data security assessments for cross-border data flows.
- Indian collaborators have fewer formal requirements but face ethical concerns about data from vulnerable populations.

For example, when a European researcher wants to share a dataset with Chinese colleagues, they must navigate incompatible requirements: GDPR requires specific consent and purpose limitation, while Chinese regulations demand government access to data and algorithms for security reviews.

*Proposed solution*: A complementary approach is the kind of “regulatory capacity building,” further described in the next section. Creating shared expertise pools through multi-jurisdictional “common capacity” hubs could facilitate harmonized standards for GLM development across borders that ensure regulatory compliance, including appropriate privacy and security protections.

### Scenario 3 Safely open-sourcing models to promote innovation

The GenePredictAI consortium trained the model on publicly available genomic datasets from population studies. The consortium is considering whether to release it publicly for anyone to use, similar to how models like BERT, LLaMA, or DeepSeek have been shared (Gibney 2022; Vake et al. 2025). By contrast, ChatGPT and Anthropic’s Claude are closed-source models that can only be used through API calls or web applications that access dedicated cloud servers. In the AI paradigm, open source releases are broadly encouraged. This approach embraces the philosophy that open access drives innovation by allowing researchers to build on each other’s work. Notably, current AI regulations primarily focus on mitigating high-risk applications rather than restricting model sharing.

The genomic regulatory paradigm, on the other hand, emphasizes tight controls on who can access genetic information and for what purpose. The black-box nature of GLMs, however, makes it difficult to audit exactly what genetic information might be embedded in model weights. Therefore, if the GenePredictAI team were to release their model weights publicly, a third party could probe those weights to identify participants and obtain sensitive health information without consent.

*Proposed solution*: The conflict between open source aims and protecting privacy can be mitigated through a combination of GLM-specific certification with technical measures., unlike Case 1, it is not enough to restrict responses to model queries to avoid downstream privacy risks. Because the model itself can be used downstream without supervision, certification must establish clear standards for model release, including requirements for privacy-preserving embeddings and transformations, as described in the Technical Approaches section below. Certification should include accountability mechanisms, to ensure that technical solutions are adequate to prevent the extractability of personal genetic information.

## The evolution and architecture of GLMs

**Fig. 1:**
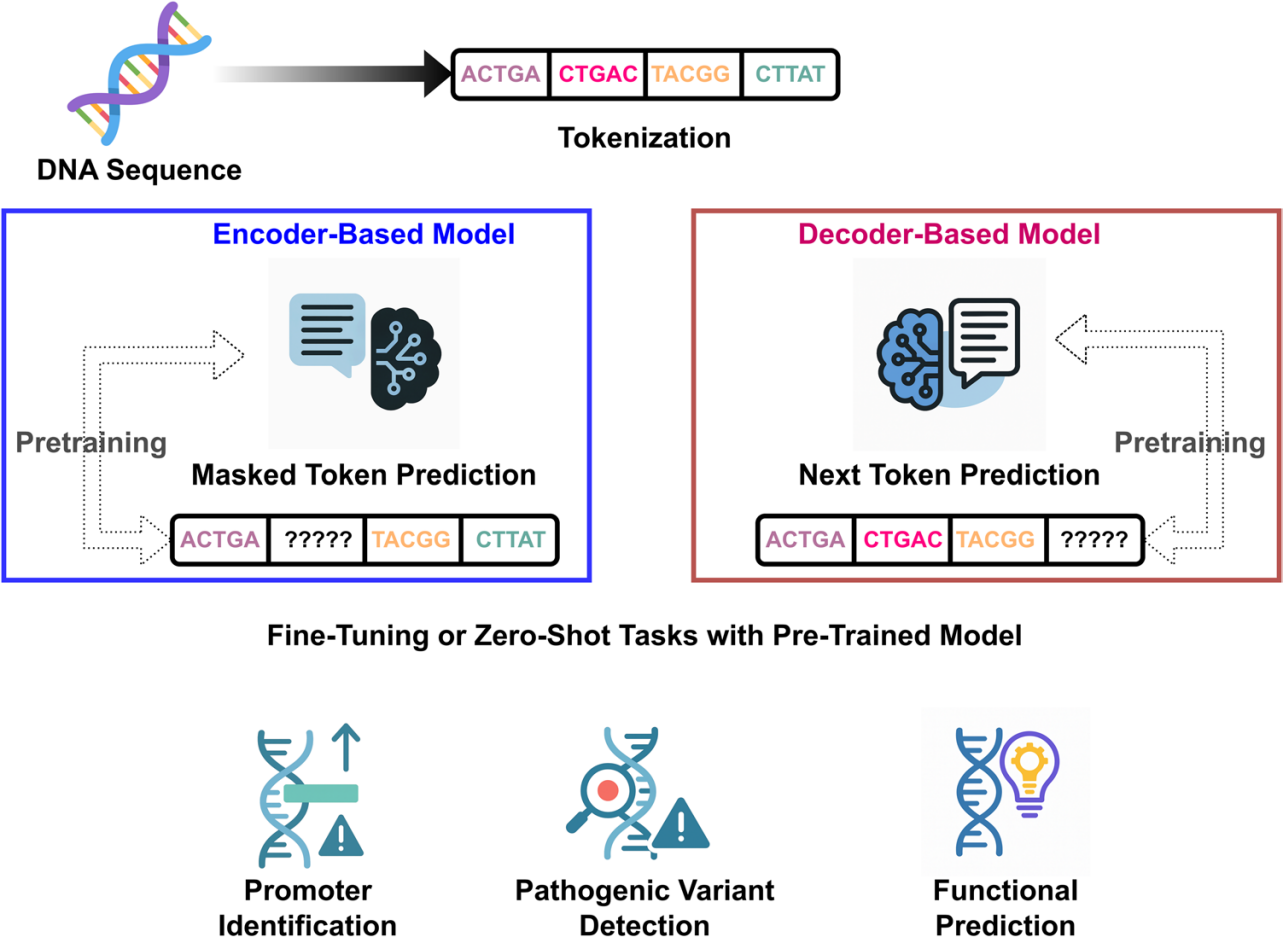
Schematic overview of genome language models (GLMs). GLMs process DNA sequences as input and convert them into smaller fragments called *tokens*. These tokens may be fixed-length *k*-mers (shown here) or generated using methods adapted from natural language processing, such as byte-pair encoding. Two alternative architectures are depicted: encoder-based models. e.g., DNABERT (Ji et al. 2021), which rely on masked token prediction during pretraining, while decoder-based models, e.g., genomic GPTs such as EVO (Nguyen et al. 2024), predict the next token in sequence. Some models are also multimodal, integrating textual annotation or other kinds of biologically relevant inputs. Both model types are pretrained on large-scale unlabeled genomic datasets and can be fine-tuned or used in zero-shot fashion for downstream tasks such as promoter identification, pathogenic variant detection, disease risk prediction, and other functional genomics applications

The first GLMs like DNABERT pioneered GLMs by adapting the BERT architecture—originally developed for human language processing tasks like translation and semantic text search—to genomic data (Ji et al. 2021; Zhou et al. 2023) (Fig. 1). DNABERT, and GLMs more generally, process split DNA sequence inputs into small fragments, called *tokens*. These can be *k*-mers, which are subsequences of constant length *k* or, in the case of “byte-pair encodings,” the fragments are of variable length determined as part of model pretraining process. DNABERT and related models, as in the original BERT, employ a “masked language modeling” objective during *pretraining*. Genomic DNA is split into subsequences, in the case of original DNABERT, 10 to 510 base sequences obtained through non-overlap splitting or random sampling. Each resulting subsequence represents an unsupervised training sample. The DNABERT model is then pretrained by masking each sample at random tokens, with the training objective being to predict the masked tokens (i.e., restore the original training sample) based on surrounding context. Pretraining the GLM on massive unlabeled genomic datasets enables the model to learn fundamental biological patterns before being fine-tuned for specific downstream tasks like promoter identification or variant effect prediction (Refahi et al. 2025; Benegas et al. 2023; Sanabria et al. 2024; Boshar et al. 2024).

Despite their interpretive limitations, the technical capabilities of GLMs have translated into rapidly expanding applications. They can predict gene expression patterns, identify regulatory elements, and detect pathogenic variants (Refahi et al. 2025; Dalla-Torre et al. 2023; Zhou et al. 2023; Nguyen et al. 2024; Leksono and Purwarianti 2017). In clinical settings, GLMs show promise for interpreting variants of unknown significance, potentially transforming genetic diagnosis and personalized medicine (Benegas et al. 2023).

Dalla-Torre et al. (2023); Nguyen et al. (2023) DNABERT is an example of an “encoder-based” model, which encodes sequences into numerical representations that can be used for classification and prediction tasks based on the full sequence context. Alternatively, decoder-based generative GLMs adapt GPT architectures used in LLMs like ChatGPT, Anthropic, and DeepSeek (İhtiyar and Özgür 2024; Nguyen et al. 2024; Consens et al. 2025). Instead of masked token prediction, these models learn to predict the “next token” in a sequence. Some decoder-based GLMs even offer multimodal capabilities that integrate, for example, text queries and information with DNA sequence processing, alignment, and generation (Jin et al. 2024; Liang et al. 2024; Asim et al. 2025). Subsequently introduced models such as HyenaDNA (which uses next token prediction), Nucleotide Transformer (an encoder-based model), and Google AlphaGenome (a custom encoder-based architecture), enable pretraining and prediction based on potentially longer sequences, through architectural innovations like state space models and efficient attention mechanisms (Dalla-Torre et al. 2023; Nguyen et al. 2023; Avsec et al. 2025).

Both encoder- and decoder-based GLMs learn from large genomic datasets spanning human genomes and variants, as well as other species, enabling these models to identify features associated with, e.g., disease risk and evolutionary patterns. A core challenge with all GLMs, however, is that—like other LLMs—they are “black boxes” with extremely complicated and vast neural network architectures. Thus, it is difficult to interpret or audit the internal logic behind their outputs, including fine-tuned predictions or newly inferred associations (Murdoch et al. 2019; Linardatos et al. 2021).

The power of GLMs raises significant regulatory challenges. These concerns mirror broader issues with foundation models, which, because of their size, scope, and potential capabilities, present numerous ethical challenges (Bommasani et al. 2021). Even more so than other AI systems, GLMs specifically work with genetic information that is uniquely personal, immutable, and has implications extending to family members and future generations. Ethicists and policymakers have identified considerable issues regarding privacy, consent, and the potential for encouraging bias and enabling discrimination through large-scale genomic analysis (Kaye et al. 2012; Bilkey et al. 2019). GLMs, however, unlike conventional genetic analysis, involve computational approaches that can infer information beyond the original scope of consent or intended use, such as patterns in genomic data that might reveal sensitive information. Comparable risks have been documented in the context of large language models (LLMs), where training on datasets containing personally identifiable information (PII) has, in some cases, resulted in models inadvertently disclosing sensitive details during generation (Aditya et al. 2024; Das et al. 2025; Lukas et al. 2023).

Generative GLMs also present biosecurity concerns, as they could potentially be used to design sequences with pathogenic properties or circumvent existing screening protocols for synthetic DNA (Mackelprang et al. 2022; Kurtoğlu et al. 2024).These capabilities have implications not only for clinical applications but also for the direct-to-consumer (D2C) genomics market, where individuals obtain genetic information, including whole genome sequences, through commercial services without clinical oversight (Samlali et al. 2022). Via D2C sequencing and publicly available of GLMs, consumers may unknowingly expose their genetic data to powerful inference capabilities without institutional protections.

Accordingly, established protocols and legal protections for genetic and health information, may not adequately address the expanded capabilities of GLMs for both beneficial innovation and potential misuse.

## Current regulatory landscapes in AI and genomics

The regulatory environment for AI is rapidly progressing, though still very much a work in progress. AI governance frameworks are rapidly emerging worldwide. Prominent examples include:

- The European Union’s AI Act, which categories AI systems based on risk levels (van Kolfschooten and van Oirschot 2024; Evas 2024). In the EU scheme, systems deemed to pose an “unacceptable risk”—such as uses for social scoring by government and cognitive manipulation—are outright prohibited. Strict requirements on data governance, transparency, and oversight are imposed on high-risk applications, including critical infrastructure, education, employment, and law enforcement. Limited-risk systems, such as chatbots, require specific transparency obligations (e.g., informing users they are interacting with AI), while minimal-risk systems face no regulatory burdens.
- China’s regulations on algorithms including draft rules on generative AI (Sheehan 2023; Franks et al. 2024). China’s draft rules emphasize data provenance, requiring the use of “legitimate data” for training, requiring that training data and outputs be “true and accurate.” Overall, China’s rules reflect a top-down regulatory ethos, with a focus on content control, ideological alignment, and national security.
- Competing Executive Orders in the United States, including the Biden Administration’s Order, which primarily focused on AI systems’ outputs, transparency requirements, and potential for discrimination or manipulation, and the Trump Administration’s de-emphasizing regulation while ensuring viewpoint diversity (Lubello 2025).

Similar AI governance efforts are being undertaken elsewhere. For example, AI policy development is widespread in Latin America; although, proposed legislation has in many cases not yet been enacted as technologies and policy considerations continue to evolve (Flórez Rojas 2025). In Canada, executive-directed policy has been developed by the Canadian Treasury Board, which created the Algorithmic Impact Assessments (AIA) mechanism. Canadian government agencies use AIA reviews to preemptively evaluate impacts, e.g., potential risk of bias or unfairness, of using algorithms on institutions, individuals, and groups (McKelvey and MacDonald 2019). Emerging economies in Africa have also been developing AI policies, investment initiatives, and regulations (Diallo et al. 2025). International bodies, such as UNESCO and the OECD, have also developed ethical guidance frameworks, aiming to build a global consensus around shared norms like human rights, transparency, and accountability (Bean et al. 2025; Yeung 2020). Many countries now cite UNESCO and OECD principles in their own AI rules (Corrêa et al. 2023). The number of national and sub-national governments, regional and international alliances, and non-governmental organizations releasing AI policy documents and developing regulations number in the hundreds, and continues to grow at a rapid pace (Corrêa et al. 2023).

As a whole, the AI policy frameworks that are emerging broadly emphasize the risk of AI use and the potential for bias and unfairness, along with requirements for transparency, mechanisms to mitigate disinformation, and protections for the intellectual property rights of creators (Schiff et al. 2020; Miazi 2023; Schmitt 2022; Batool et al. 2025). These measures aim to ensure both accountability and trust in the deployment of AI technologies, especially in high-risk contexts like healthcare, law enforcement, and employment.

Conversely, genomic regulations center on issues of privacy, non-discrimination based on personal information, and informed consent, reflecting the deeply personal and potentially predictive nature of genetic data. For example, in the United States, the Genetic Information Nondiscrimination Act (GINA) prohibits discrimination in health insurance and employment based on genetic information, though it does not extend to life or disability insurance (Roberts 2010; Joly and Dalpe 2022; Suter 2019). Meanwhile, the Health Insurance Portability and Accountability Act (HIPAA) provides specific privacy protections for health data, including genetic information, and requires patient authorization for disclosure in most circumstances (Cohen and Mello 2018; Marchant et al. 2020). Canada’s Genetic Non-Discrimination Act similarly restricts requiring genetic testing or disclosure in contractual contexts, with criminal penalties for violations (Di Felice 2024). Europe’s General Data Protection Regulation (GDPR) regulates direct-to-consumer genetic testing by explicitly recognizing genetic data as a special category requiring heightened protection, thereby limiting its processing except under strict conditions, such as explicit consent or substantial public interest (Buiten 2021; Harbord 2019). In China, the genetic diagnostic testing industry has struggled between a lack of applicable laws and regulation leading to an emphasis on self-regulation and potential risks to individuals of loss of privacy. It appears as though this has paradoxically restricted growth of the industry in China that could have occurred if there were more certain rules (Yichao et al. 2023; Du and Wang 2020).

Notably, even in the United States, which has robust non-discrimination and privacy statutes and enforcement mechanisms, the policies around genetics still fail to address a number of critical circumstances. One significant gap is that in private transactions, there is a presumption that consumer consent can override government regulations, even when that consent may lead to outcomes that a nonexpert would not foresee. For example, recently one of the pioneering direct-to-consumer genetic testing companies, 23andMe, went bankrupt. Because it was a consumer test, HIPAA did not necessarily apply. As a result, customers who had not downloaded and deleted their information prior to bankruptcy faced the possibility of not only losing their data but also having it sold to others (Drabiak 2017; Kwon 2025). Consumers had consented to share their data but did not anticipate the risk of a seemingly well-financed, heavily advertised company going out of business.

An important drawback of the GINA and HIPAA frameworks is that they are based on preventing the request for and disclosure of genetic information. Legal provisions that focus on non-disclosure may hinder positive measures to support individuals with genetic predispositions and diseases that do not manifest in ways that fall into protections under disability law (Roberts 2011). For instance, GINA, HIPAA, and similar frameworks may inadvertently block measures that could address racial and socioeconomic disparities in disease risk. Indeed, when race factors have been excluded from studies in a well-intentioned effort to control, it has sometimes backfired. For example, studies of colorectal cancer prediction and healthcare allocation models have shown that removing race data adversely affected minority, specifically Hispanic, patients. Nominally “unbiased” models failed to account for the higher disease prevalence in their communities, leading to insufficient resource allocation (Khor et al. 2023; Ledford 2019; Obermeyer et al. 2019). As further explained in the next section, GLMs exacerbate these regulatory gaps and reinforce the need for creative, specifically tailored policy and technical measures.

## Regulatory gaps at the intersection of AI and genomics governance

By combining the expansive inference capabilities of generative AI with sensitive genomic data, GLMs create unprecedented policy tensions across multiple dimensions—privacy, consent, bias, interpretability, and jurisdictional compliance. Table 2 summarizes these tensions and the regulatory challenges they generate, as further explained in this section.

**Table 2:** Key regulatory contrasts and challenges for GLMs

Dimension	AI regulations	Genomic regulations	GLM-specific challenges
Data Privacy	Reliance on anonymization generally sufficient	Genetic data inherently identifiable, even after de-identification	Inference capabilities undermine anonymization
Consent Models	Broad or general consent typically accepted	Specific, purpose-limited informed consent typically required	Generative insights exceed original consent scope
Bias and Fairness	Focus on algorithmic bias related to observable proxies	Need to carefully consider addressing subgroup fairness or systemic bias	Amplifies biologically embedded biases
Interpretability	Black-box models accepted in some domains	High explainability required in clinical genomics	GLMs lack interpretability needed for clinical trust
Jurisdictional Boundaries	AI development and models often cross international borders	Strict national limits on, and control over, data sharing and use	Cross-border training and inference complicate compliance
Legal Liability	Liability frameworks emerging, typically placing accountability on users interpreting outputs	Clear liability tied to clinical standards and regulatory compliance	Unclear division of responsibility among developers, providers, and deploying institutions

GLMs operate at the intersection of AI and genomics, inheriting different kinds of ethical and policy considerations from both domains while introducing new tensions that neither framework full addresses, as summarized here

### Data privacy and informed consent

GLMs present new challenges to existing privacy- and consent-based frameworks for genetic studies. Because they operate at genomic scale, GLMs raise concerns about the identifiability of individuals whose sequences are included in genomic datasets—a critical issue since large-scale genomic technologies emerged in the 2000s (Lowrance and Collins 2007). Empirical work shows that even minimal genotype windows can localize an individual to within 100 km (Battey et al. 2020), challenging the assumption that anonymized DNA poses low risk. Trait-prediction studies demonstrate that GLMs can regenerate quasi-biometric data such as facial structure from whole genome sequencing data (Lippert et al. 2017), further complicating privacy. Unlike traditional genetic studies that test prespecified markers with known outcomes, GLMs function as generative inference engines, uncovering novel, unexpected associations without explicit programming or human direction.

Pretraining on massive genomic datasets enables GLMs to capture large amounts of data within model weights. Somewhat paradoxically, the embedding of data is too complex to readily censor problematic information in a systematic way; yet, it may still be possible to extract such information, whether intentionally or not. In the LLM context, this is referred to as the risk of personally identifiable information “leaking” in large language models (Aditya et al. 2024; Das et al. 2025; Lukas et al. 2023; Yao et al. 2024). While conventional statistical approaches to de-identification have been applied to genome databases (Bonomi et al. 2020; Thomas et al. 2024), in practice they are insufficient for GLMs, which can infer connections across seemingly unrelated genetic markers that conventional statistical methods miss. For example, a recent review found that secondary findings in hereditary cancer genes—i.e., findings unrelated to the primary indication for the genetic test—occur in approximately 0.4 to 3.1% of cases (Avsec et al. 2025). Given that GLMs operate at a much larger scale across the genome than conventional genetic tests, it is likely that unexpected findings will emerge as systematic and automatic outputs. These findings, in turn, raise the challenging question of whether and how to inform individuals about unanticipated but significant genetic risks.

The use of GLMs thereby complicates existing regulatory regimes governing genetic data. For instance, while anonymized data is not covered by the EU’s principal digital data protection law (the GDPR), de-identified genetic data used by GLMs can contain enough information to allow re-identification—fewer than 100 single nucleotide polymorphisms could uniquely identify individuals (Shabani and Marelli 2019). To be sure, the GDPR takes a contextual approach to anonymization and could treat genomic data as personal data if re-identification is reasonably possible, but that remains a high threshold to meet. The HIPAA framework relies on a fixed list of identifiers to determine whether data is de-identified, and, thus, it may also not apply effectively to GLMs since it potentially overlooks re-identification via advanced genomic analysis. More broadly, informed consent frameworks, whether statutory (HIPAA and GDPR) or contractual (such as those used for direct-to-consumer testing), presume participants can meaningfully consent to specific, foreseeable uses of their data. GLMs undercut these basic assumptions. GLMs undercut this assumption. They can autonomously reveal new and unanticipated insights from existing genomic data, potentially revealing information that was not extractable at the time consent was given.

Further complicating matters is legal ambiguity over whether and how findings that emerge from GLMs should be disclosed to third parties, especially family members. So-called “Traceback” programs are genetic testing initiatives where, when a proband (identification of pathogenic variant) has been found, relatives are notified about their potential risk and invite them for testing as well. However, HIPAA's Public Health Exception cannot currently facilitate Traceback programs) due to inconsistent state laws and genetic privacy statutes (Wagner et al. 2022). GLMs amplify this regulatory challenge. Unlike traditional, targeted genetic analyses, GLMs can autonomously identify patterns across entire genomes, thereby potentially affecting thousands of families simultaneously through unexpected inferences about disease risks, carrier status, and complex multigenic traits, even when they are not specifically instructed to search for them. Troublingly, GLMs, like other generative AI models, can produce hallucinations (Xu et al. 2024) that result in erroneous predictions with potentially serious consequences for individuals and families receiving genetic risk information.

In sum, the unique properties of GLMs as compared to other machine-learning based genetic analysis creates an entirely new category of privacy and misinformation concerns that existing frameworks, designed for more limited and directed genetic analyses, cannot adequately address.

### Bias and fairness

AI policies increasingly aim to mitigate algorithmic bias through transparency, fairness audits, and data governance, where they typically focus on observable outputs and proxy variables, such as race, gender, or socioeconomic status (Brown et al. 2021; Groves et al. 2024; O’Neil et al. 2024). However, GLMs, like any generative AI—or, indeed, any kind of machine learning models—can only be as unbiased and fair as the data they are trained on (Lenders and Oloo 2024; Ayoub et al. 2024). GLM outputs will thus invariably reflect any content bias present in their training data, given what has been observed for LLMs more generally (Acerbi and Stubbersfield 2023). The specific challenge with GLMs, however, is that unlike LLMs more generally, bias in genomics and health can be more deeply embedded and biologically entangled.

In conventional AI systems, misclassifications can often be traced to proxy variables like cost or geographical identifiers such as the postal (ZIP) code (Obermeyer et al. 2019). GLMs, though, may produce biased predictions due to patterns that could evade fairness audits that focus only on easily identifiable proxies. Even if an effort is made to train GLMs on comprehensive, population-scale datasets, such datasets may nevertheless be unevenly distributed, i.e., often skewed toward populations of European ancestry (Spratt et al. 2016). Such a model trained on such an inherently biased genomic data set may end up leading to clinical deficiencies, e.g., unable to identify significant variants associated with disease risk or therapeutic efficacy in underrepresented populations (Martin et al. 2017). As described above, the GINA and HIPAA paradigms of genetic privacy and absolute non-discrimination can exacerbate these issues, by preventing the identification of bias in training data for GLMs. Moreover, the black box nature of GLMs makes it difficult to apply statistical adjustments to mitigate bias, including standard methods like subpopulation calibration (Barda et al. 2021).

### Interpretability, explainability, and transparency

Interpretability, explainability, and transparency have been proposed as key pillars of AI policy that can help identify potential bias and ensure the reliability of decisions made based on AI models (Lahusen et al. 2024; Balasubramaniam et al. 2023). However, this is another dimension where there is a gap between AI governance principles and essential considerations for genetics and biomedicine.

While the transparency of initial pretraining data can help identify potential areas of bias, in the case of GLMs, even publicly available genomic data may be inherently biased due to uneven distributions of samples across populations (Kessler et al. 2016; Günther and Nettelblad 2019; Fatumo et al. 2022). The high cost of obtaining quality data means that assembling a properly diverse dataset can be prohibitively expensive or practically infeasible at the time of training. Even when datasets are supplemented by additional sampling outside of public databases, transparency requirements can conflict with privacy concerns. While data bias can be mitigated by including additional metadata associated with samples, the more metadata is known about genetic data, the greater the risk of extracting identifiable information associated with individuals—further compromising individual privacy. For example, even where data have been fully deidentified, one frequently cited 2019 study found that the identity 99.98% of Americans could be recovered using 15 demographic attributes (Rocher et al. 2019). Therefore, while it can help with identifying potential areas of bias and ultimately reducing them, transparency of training data at the same time may lead to other deleterious impacts on individuals. This has practical implications, because such transparency is a critical part of AI regulation in many jurisdictions, such as China and Europe.

The challenge of model explainability and interpretability raises further tensions between AI and biomedical policy. AI governance frameworks often emphasize interpretability; however, there is also active debate regarding the degree to which, especially for certain lower-stakes applications (e.g., recommendations), peak performance and usability might justify less explainability, as full transparency could overwhelm users (Mann 2023). Additionally, most explainability techniques for LLMs are limited to *post-hoc* explanations (generated after model inference), since building inherently interpretable models is technically unfeasible at present (Wang et al. 2023; Gurrapu et al. 2023).

However, there are compelling arguments that post-hoc explanations applied to “black boxes,” like GLMs, are fundamentally unreliable approximations (Rudin 2019). Cynthia Rudin’s research shows that such explanations lack fidelity to the actual model’s computations, potentially masking critical underlying issues. For example, models can rely on spurious data artifacts. In one documented case, a chest X-ray model appeared to identify pneumonia but was actually detecting the word “portable” embedded in X-ray images taken with portable machines, which are more commonly used for sicker patients (Zech et al. 2018). This kind of flawed explanation creates what has been called an “ersatz understanding” of an LLM: one that does not actually provide insight into how it works and therefore cannot provide a basis to justify the model’s conclusions (Babic et al. 2021).

Limits on model explainability are a major challenge in biomedicine, where clinical trust is critical. Clinicians require insight into a model's reasoning not just for trust, but for clinical actionability and safety (Petch et al. 2022). A prominent example of where things can go wrong is the Epic Sepsis Model, a widely-used system that predicts sepsis risk but provides little explanation for its alerts—and when conditions changed, resulted in erroneous predictions that could not be understood because of the lack of transparency and explanation (Wong et al. 2021; Zhang et al. 2024). GLMs present additional complexity beyond general AI systems, because there are so many potential explanatory targets: what aspects of the system or the model are leading to results, how to control for false discovery error rates, and at what level of granularity to identify features that drive predictions (Watson 2021). When researchers apply standard machine learning explanation techniques to genomic data, it can be very hard if not impossible to distinguish between explanations due to bias factors or legitimate biological factors (Momenzadeh et al. 2022). Therefore, the challenge of GLM interpretability creates another critical governance issue that neither current AI nor genomic approaches fully address.

### Jurisdictional complexity and uncertain liability frameworks

The global nature of AI development and cloud-based inference allows models and datasets to technically flow across national boundaries without physical impediments, allowing data from around the world to be aggregated and disseminated broadly. The free flow and aggregation of data across jurisdictions complicates regulatory compliance. (Cheng 2025; Mickle et al. 2025) Previously, differences in genetic privacy laws across countries had limited practical implications for patients receiving clinical care from providers who conduct and use the results of genetic testing, or other individuals who might be seeking health information more generally. Ordinarily, health information directed to individuals typically remained confined within national or subnational borders and institutional contexts. This allowed different countries to maintain different regulatory regimes.

Cloud-based AI systems fundamentally change this landscape. For example, when DeepSeek, a powerful LLM based in China, emerged, it immediately led to national security concerns in the US around users potentially sharing data with China-based cloud servers (Cheng et al. 2025; Mickle et al. 2025). Cloud computing and transborder data flows create new transnational governance problems (Arner et al. 2022; Floridi 2020). A GLM trained in a jurisdiction with minimal genetic privacy protections—whether due to regulatory gaps or lax enforcement—could violate regulations when deployed in jurisdictions with stricter privacy frameworks. Inadvertently using a GLM from a laxer jurisdiction could result in considerable compliance risks and uncertainty for researchers, developers, and healthcare providers.

Notably, in the area of genomics *research*, international collaborations have successfully operated across borders, such as the International Cancer Genome Consortium (ICGC) and the Global Alliance for Genomics and Health (GA4GH) (Hudson (Chairperson) et al. 2010; Global Alliance for Genomics and Health 2016). However, their focus is narrower—focusing on research rather than healthcare and providing health information to individuals. Because they are directed to research, international collaborations are typically between well-established and highly regulated academic and governmental institutions. As a result, they can operate through formal and enforceable schemes that include structured governance, data-access committees, standardized consent frameworks, and controlled-access repositories (Knoppers and Joly 2018). Any liability that might arise in the course of such projects is clearly attributed to entities operating within the patient’s jurisdiction. By contrast, for GLMs, information may be generated in one jurisdiction but employed to provide healthcare in another, creating potential jurisdictional conflicts.

Beyond jurisdictional uncertainty, the rules around legal liability for GLMs remain unclear. When GLMs generate outputs that directly impact clinical decisions or insurance assessments, any harms that arise may incur liability. It remains unclear, however, whether legal responsibility for those harms should be attributed to model developers, the healthcare providers interpreting model outputs, or the institutions who deploy these technologies (Cestonaro et al. 2023). The use of LLMs in clinical contexts is still in its infancy. Accordingly, the extent of physician and model provider liability for the use of AI in clinical practice remains in fluxl (Mello and Guha 2023; Shumway and Hartman 2024). Progress on legislation has been limited. Draft legislation was proposed in the European Union, but, after years of deliberation, it was withdrawn in early 2025 (da Fonseca, Vaz de Sequeira, and Barreto Xavier 2024; Andrews 2025). A bill has also been proposed at the state level in California that would limit liability for model developers who were able to certify adherence to best practices for model safety. But as of mid-2025, the bill has stalled (McNerney 2025).

The future of medical liability for LLM use may parallel the practice of law, where LLMs are already being used. In law, there is an emerging consensus where courts hold attorneys accountable for misusing LLM, such as submitting legal arguments that include hallucinated citations to non-existent cases, on the basis that attorneys can reasonably be expected to verify the AI-generated output (Lent and Paek 2024). Whether there is actual *malpractice* liability, that is, whether attorneys are liable to clients in cases of AI misuse, as opposed to the model providers, however, is a more complicated and still unsettled question (Johnson 2024). Determining liability for AI use in the biomedical sector is likely to involve similar considerations, given that the clinician’s responsibility, like that of an attorney, is considered to be paramount.

There are many considerations as to where liability should lie for use of AI in genetic diagnostics, and the path forward is not yet certain. For example, is there liability if an individual receives inaccurate health information from a model, even when the response is accompanied by a generic disclaimer? Does the model provider have an additional responsibility to specifically censor medical information from outputs? Relevantly, one study has shown that efforts to prevent health misinformation from being output from general-purpose LLMs were inconsistently applied even when model developers were made aware of risks (Menz et al. 2024). These liability issues mirror that of the use of AI in clinical medicine more generally, where there is uncertainty and active debate on allocating fault for medical malpractice connected to AI (Cestonaro et al. 2023).

Policy recommendations that have been proposed for addressing AI for medicine more broadly include mandating FDA oversight for clinical AI validation, requiring algorithmic transparency with peer-reviewed data support, and implementing liability reform that appropriately distributes responsibility between physicians and AI developers (Shumway and Hartman 2024). However, the lack of adoption of any of these recommendations, or even any concerted effort to address AI medical liability issues, underscores the urgency of developing innovative frameworks to address unique liability challenges posed by GLMs.

The applications of GLMs in clinical practice present additional complications. Interpreting genetic data (such as raw DNA sequence variants) requires specialized expertise, making it considerably more difficult for healthcare providers to verify predictions thoroughly. Furthermore, one of the central promises of GLMs lies in improving clinical efficiency and efficacy by automating complex analyses. Expecting healthcare providers to carefully validate each instance and detail of a GLM’s predictions may thus be both unrealistic and counterproductive. Accordingly, the absence of clear legal frameworks defining responsibilities and liabilities for GLM-generated outputs is a gap that needs to be filled for GLMs to be effectively used in clinical contexts.

## Bridging the gap: integrative GLM governance

Addressing the regulatory challenges created by GLMs requires integrative governance approaches that bridge AI and genomics. We propose a layered regulatory model that establishes overarching principles applicable to all GLMs, while allowing for context-specific requirements based on risks arising from specific application risk and varying levels of sensitivity of different categories of data. The regulations that arise from a layered model thereby enable appropriate protections without imposing unworkable restrictions on beneficial research and applications.

**Fig. 2:**
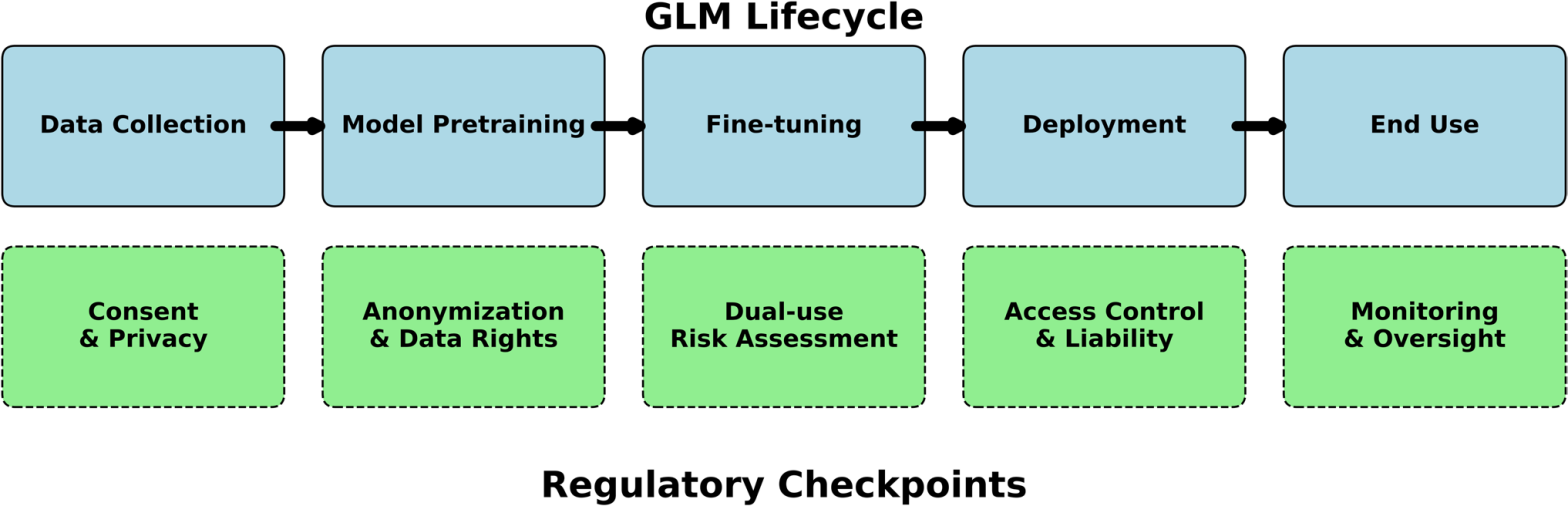
In a multilayered governance approach to GLMs, the sequential stages in the development and deployment of models are aligned with critical regulatory intervention points. Key oversight areas correspond to each stage of GLM development. Their alignment emphasizes where current governance frameworks must adapt or expand to effectively manage the risk and responsibilities associated with GLMs

Figure 2 illustrates key intervention points where layered governance measures can be applied throughout the GLM lifecycle, from initial data collection through deployment and application of GLMs. These intervention points align regulatory checkpoints with each stage of development, emphasizing where current frameworks must adapt to effectively manage the unique risks associated with GLMs.

**Fig. 3:**
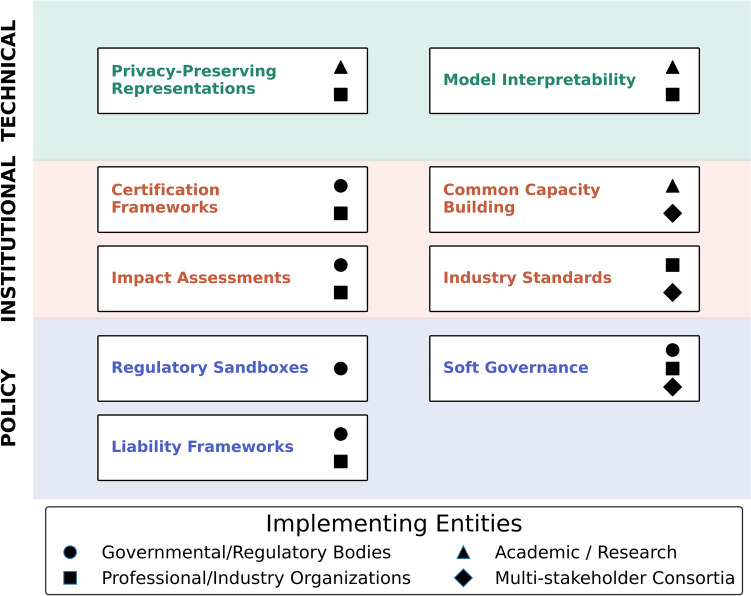
This diagram illustrates the proposed multilayered governance framework for Genome Language Models (GLMs). The framework comprises technical, institutional, and policy layers. The institutional layer bridges between technology and policy, where the technical layer helps support and implement policy, while policy enables and regulates technology. The types of organizations responsible for implementing each element are also indicated, as many governance mechanisms require collaboration across multiple actor categories

Figure 3 presents a proposed multilayered governance framework, highlighting how technical solutions, institutional mechanisms, and policy frameworks interact to create a comprehensive approach to GLM oversight. This layered structure recognizes that effective governance requires complementary measures at different levels, from privacy-preserving data representations at the technical layer to regulatory sandboxes at the policy layer. There is an important role for both policy and technical measures, examples of which are provided below in turn.

### Policy approaches

Addressing the regulatory challenges created by GLMs requires a comprehensive policy framework that operates at multiple levels. The following proposed components—regulatory sandboxes, soft governance mechanisms, capacity building, and certification frameworks—are each discussed in turn below. Together, they address distinct aspects of the GLM governance challenge, from enabling controlled testing environments to establishing accountability mechanisms. These proposals can work in concert to build adaptive oversight structures suited to the rapid evolution of GLM technology.

#### GLM-specific regulatory sandboxes

A regulatory sandbox is a controlled environment established by regulators that allows firms to test innovative technologies with regulatory flexibility and direct supervision for a limited period of time (Yordanova and Bertels 2024; OECD 2023). Regulatory sandboxes enable testing balances between innovation and safety considerations (Buocz, Pfotenhauer, and Eisenberger 2023), such as, in the case of GLMs, reliance on broad data access with stringent privacy and consent requirements. Regulatory sandboxes further enable evaluating technical standards, such as for privacy preservation, fairness auditing for bias, or model interpretability benchmarks (Leckenby et al. 2021). Designing effective and safe GLM sandboxes will require confronting practical implementation challenges, as further detailed below.

Implementing GLM-specific sandboxes will require coordinated effort across multiple stakeholders. At the national level, primary responsibility could rest with existing regulatory bodies such as health authorities, environmental agencies, or cross-governmental innovation offices, depending on the specific application domain (Johnson 2024) Defining eligibility for the sandbox is particularly important to ensure fairness and prevent bias, promote regulatory neutrality and trust, and help regulators manage risk and achieve the objectives of evaluating potential regulatory structures (Gumbo and Chude-Okonkwo 2025; OECD 2023).

Moreover, the funding model of GLM regulatory sandboxes can be adapted from the fintech (financial technology) sector, where regulatory sandboxes in many countries were found to be empirically successful in increasing investment while reducing regulatory uncertainty (Goo and Heo 2020). Funding models from fintech sandboxes include combining government seed funding with cost-recovery mechanisms through application fees and graduated licensing structures (Goo and Heo 2020).

GLM sandbox design demands broad expertise, since it involves deep, multidisciplinary knowledge that covers AI, genomics, as well as healthcare or other application domains. At the same time, it is important to avoid conflicts of interest that can compromise safety within the sandbox and bias the interpretation of outcomes (Brown and Piroska 2021). Based on successful sandbox models in other sectors, GLM sandboxes can be made effective where they specifically leverage distinct strengths of the more neutral academic and professional community along with the application-oriented industry. Specifically, GLM sandbox design should rely on (i) professional bodies and academic institutions for technical expertise and independent oversight, and (ii) industry associations for standard-setting and peer review.

International coordination is also particularly important for GLMs, given the cross-border nature of genomic data, model development, and model hosting. Coordination can be addressed by bodies like the WHO or OECD, who can facilitate the harmonization of standards and mutual recognition agreements. Regular multilateral expert working groups can be convened that include representatives from both AI and genomics communities to ensure standards remain current with technological advances.

Additionally, GLM sandboxes face unique challenges around the boundaries of consent. Unlike conventional AI systems where sandbox participation can be limited to consenting users, GLMs trained on genomic data inherently affect non-participants. Even minimal genomic data can identify individuals *and* their relatives (Battey et al. 2020; Wagner et al. 2022), extending impacts through family networks beyond an individual who has agreed to use a GLM within a sandbox. This presents a practical governance problem, on top of the existing issues with informed consent in the general AI context, which has been a point of critique in the EU AI Act sandbox proposals (Buocz, Pfotenhauer, and Eisenberger 2023). Regulators can address these concerns by designing GLM sandboxes to:

- Mandate federated learning approaches where a shared model is trained while genomic data are kept on local computers without sharing (Zheng et al. 2024);
- Require "genetic impact assessments,” patterned on the AIA mechanisms described above, that map potential effects on relatives and ethnic groups;
- Establish independent ethics boards to evaluate whether public benefits justify impacts on non-consenting parties;
- Include mandatory reporting on impacts external to the sandbox in exit documentation; and
- Establish a clear liability regime to redress potential harms arising during experimentation within the sandbox.

Finally, the most critical challenge comes after the regulatory sandbox ends: scaling up*.* Policymakers must recognize the difference between the temporary regulatory flexibility of the sandbox and permanent policy relaxation, especially in the face of potential pressure from financially interested stakeholders. What might have worked in the sandbox context may not translate if the relaxed rules in a sandbox are simply transferred wholesale to the national or global level. For example, a sandbox might allow genomic data sharing between a hospital and an AI firm using broad, one-time consent. Outside that context though, it could enable re-identification in ways that could engender public backlash, such as by allowing the use of genomic data to identify and arrest relatives in criminal investigations or reveal sensitive health conditions. Rather, the primary value of a GLM regulatory sandbox will lie in providing evidence-based insights to inform the development of more comprehensive and adaptive regulatory frameworks for GLMs, addressing the “pacing problem” where technology often outstrips legislative capacity (Yordanova and Bertels 2024). Accordingly, findings from the sandbox must be generalized with caution.

#### Soft governance mechanisms

“Soft governance” is a term that covers a wide range of ethical frameworks, professional standards, and industry self-regulation. These can complement formal regulations by providing flexible guidance that adapts more rapidly to technological developments (Gianni et al. 2022). One kind of soft governance mechanism are documents that are published under the authority government agencies, but which provide only voluntary guidance rather than enforceable regulation. Examples of government-issued yet voluntary guidelines are the United States’ National Institute of Standards and Technology (NIST) AI Risk Management Framework (NIST 2023) and Singapore’s Model AI Governance Framework (Allen et al. 2025). Similar soft governance frameworks can be developed through multistakeholder initiatives involving researchers, clinicians, patients, ethicists, and regulatory experts with the aim of generating consensus standards for responsible GLM development and deployment. Guidelines produced in this bottom-up, rather than top-down manner can be more responsive to technological advances and flexible to change, as compared to laws and formal government regulations which can be much slower to adapt.

However, it is important to recognize that voluntary guidelines can be overly abstract and fail to incorporate any robust mechanisms for accountability—in addition to not being enforceable. One concern is that such measures may even merely serve as “ethics washing” to preempt or delay more stringent regulation (Hao 2019; van Maanen 2022). Such approaches may inadequately address power asymmetries between developers and affected communities, or fail to sufficiently ground themselves in human rights principles (Fukuda-Parr and Gibbons 2021). For GLMs specifically, some of the risks are sufficiently harmful as to preclude merely voluntary guidelines, such as the need for strict protection of privacy, discrimination concerns, and the need for clinical trust.

Another soft governance mechanism applicable to GLMs, which has already been used for open-source AI models, is the inclusion of behavioral restrictions in licenses and terms of use (Contractor et al. 2022). Providers of open-source AI models themselves seek to regulate users through license terms that prohibit different kinds of misuse (Guerrini et al. 2017). However, licensing-based governance may not be effective. When models are open-sourced to promote transparency, they can also be replicated on servers beyond the control of the original provider. Moreover, restrictive AI model licenses may not be legally enforceable due to a fundamental flaw: The weights of the model—the components crucial to generating model output—may not be protectable intellectual property, meaning that there is nothing to license and no legal mechanism to restrict use (Cui and Araujo 2024; Henderson and Lemley 2024). This issue is perhaps even more salient for GLMs than LLMs more generally, as GLMs are trained on and generate scientific information and data, which are less likely to be subject to copyright than narrative text.

Therefore, while valuable for fostering norms and best practices, soft governance mechanisms for GLMs likely need to be complemented by clearer oversight structures, potentially incorporating elements of co-regulation, third-party auditing. This can include certification procedures, as further described below, as well as “enforceable soft law” involving voluntary commitments backed by meaningful sanctions (Han et al. 2022), ultimately ensuring that flexibility does not come at the cost of fundamental rights and public safety.

#### Regulatory capacity building

Existing institutional mechanisms for developing regulations will often lack the necessary multidisciplinary knowledge to design effective AI policy (Aitken et al. 2022). The need to establish greater capacity, including knowledge and expertise, to develop regulations is particularly acute in the case of GLMs, which require particularly specialized technical knowledge, clinical expertise, and knowledge of health care management and policy issues. GLMs sit at the intersection of two rapidly changing areas of technology: both AI and genomics. As such, a critical prerequisite for designing effective GLM regulation is the ***capacity*** to do so. Regulatory capacity is defined as a governmental agency or non-governmental organization’s ability to formulate, implement, and enforce rules effectively (Cunha and Lodge, n.d.; McAlister et al. 2024).

Furthermore, at the international level, harmonization is critical for establishing baseline standards that prevent regulatory fragmentation, particularly as data and models can so readily cross borders (Klein and Patrick 2024). Practical implementation can involve shared expertise pools or dedicated “common capacity” hubs serving as neutral sites for centers of excellence. The AI and Regulation Common Capacity Hub (ARCCH) proposal by the Turing Institute in 2022 exemplifies such an approach: an independent multi-disciplinary body that can conduct risk mapping, develop shared tools for technology evaluation, and cooperatively develop standards that can be shared globally (Aitken et al. 2022). The AI Standards Hub (https://aistandardshub.org) has emerged from that proposal with a focus specifically on developing and sharing AI standards information, representing a core component of broader capacity building (Barrance et al. 2022). Hub-based approaches for shared capacity building, while conceived for AI generally, are particularly relevant for GLMs. International cooperation is critical to ensure regions that are less powerful economically can participate equally in policy development.

To ensure continuous improvement and adaptation hubs should implement ***feedback loops*** through mechanisms including other proposals discussed here:

- Regularly convening developers, clinicians, patient advocates, and regulators to share real-world implementation experiences;
- Standardized incident reporting systems for GLM-related issues that feed into updated guidance;
- Iterative cycles based on reviews that synthesize lessons learned from regulatory sandbox experiments, certification processes, and real-world developments update soft governance guidelines.

For example, if multiple institutions report challenges with consent procedures for GLM-derived incidental findings, this would trigger a working group to develop updated consent templates and best practices. In return, the institutions will review, test, and evaluate implementation. Such an iterative process ensures that governance frameworks remain responsive to technological advances while building collective knowledge across the GLM community. Well-designed feedback loops flow bidirectionally through the layers shown in Fig. 3: practical experiences from the technical and institutional layers inform policy adjustments, while policy developments cascade down to refine certification standards and technical requirements.

#### Certification frameworks

Certification frameworks have been proposed and used in the AI policy space to evaluate models against standards addressing both AI system quality and genetic data protection, potentially increasing trust while providing clearer guidance to developers (Cihon et al. 2021). One approach is to adapt algorithm audits, such as the AIA developed in Canada, which can be adapted specifically to GLM challengeswhile collecting data on its real-world outcomes (Brown et al. 2021; McKelvey and MacDonald 2019). (Brown et al. 2021). Another approach, exemplified by Singapore's AI Verify, is to provide toolkits and processes for entities to technically test and demonstrate compliance with voluntary principles outlined in frameworks like their Model AI Governance Framework (Allen et al. 2025). Certification can enforce standards where withdrawal of certification or publicly reporting a failed audit leads to tangible consequences, for noncompliance, e.g., reputational damage or market access limitations. Certification coupled with descriptive and standardized product labeling has been found empirically to lead to more user trust and adoption, particularly where implemented by a neutral third party (Scharowski et al. 2023). Labeling could be particularly valuable for GLMs, where clinician and patient trust directly impacts the beneficial adoption of technology.

GLM-specific certification bodies could be established in different ways. To be legitimate, however, technical expertise, regulatory power, and stakeholder representation must be appropriately balanced. At the national level, taking the United States as an example, existing regulatory agencies like the FDA (for clinical applications), Federal Trade Commission (for consumer protection), or specialized AI oversight bodies could expand their mandate to include GLM certification. Professional organizations such as the American College of Medical Genetics and Genomics (ACMG) or equivalent international bodies could develop certification standards in partnership with AI governance organizations. A consortium model could be adapted for GLMs, such as one similar to the GA4GH, which was set up to develop technical standards and policy frameworks for responsible genomic data sharing across international borders (Global Alliance for Genomics and Health 2016).

Notably, while GA4GH focuses on harmonizing data formats and access protocols for traditional genomic research, a GLM-specific consortium would need to address the provision of AI-informed outputs that may be used for patient care and to inform individuals. Accordingly, such a regulatory consortium would need to develop model architecture standards, privacy-preserving training protocols, and continuous monitoring requirements. To do so, consortia will need to bring together regulatory agencies, academic institutions, and industry stakeholders to develop certification frameworks that balance innovation with safety.

Applying these frameworks to GLMs will present unique challenges. The dynamic nature of GLMs, which can evolve through ongoing training or fine-tuning, necessitates continuous monitoring and reassessment mechanisms beyond static, point-in-time certification to ensure standards are consistently met. Furthermore, the technical complexity and opacity of large foundation models underlying GLMs make thorough auditing difficult, requiring specialized expertise and potentially new standards for interpretability and explainability tailored to biological sequence analysis. Effective implementation requires overcoming hurdles such as developing consensus on GLM-specific technical and ethical standards, ensuring the availability of qualified, independent auditors, and achieving international alignment to prevent fragmentation.

### Technical approaches

While policy approaches provide essential governance frameworks, they must be complemented by concrete technical solutions to address GLM-specific challenges. Technical solutions can address both privacy and interpretability concerns in genomic language models. First, privacy-preserving data representation methods can supplement traditional anonymization by transforming genetic information into formats that maintain utility while minimizing disclosure risks. Second, advanced model architecture designs can enhance inherent interpretability, directly addressing the fundamental limitations of *post-hoc* explanation methods discussed earlier. These approaches work synergistically with the policy mechanisms described above, providing the technical infrastructure needed to implement governance principles in practice. Accordingly, the technical approaches, detailed here in turn, offer pathways to responsible genomic AI that balance analytical power with ethical considerations.

#### Privacy-preserving genomic data techniques

Privacy-preserving scoring systems represent a crucial innovation for enabling clinical utility while maintaining patient confidentiality, serving as intermediaries between raw genomic data and actionable health insights. For example, in the D2C space, one proposed methodology computes a polygenic risk score using a privacy-aware application that matches trait information to SNPs and can compute thereby privately calculate risk scores (Sandoval et al. 2023).

**Fig. 4:**
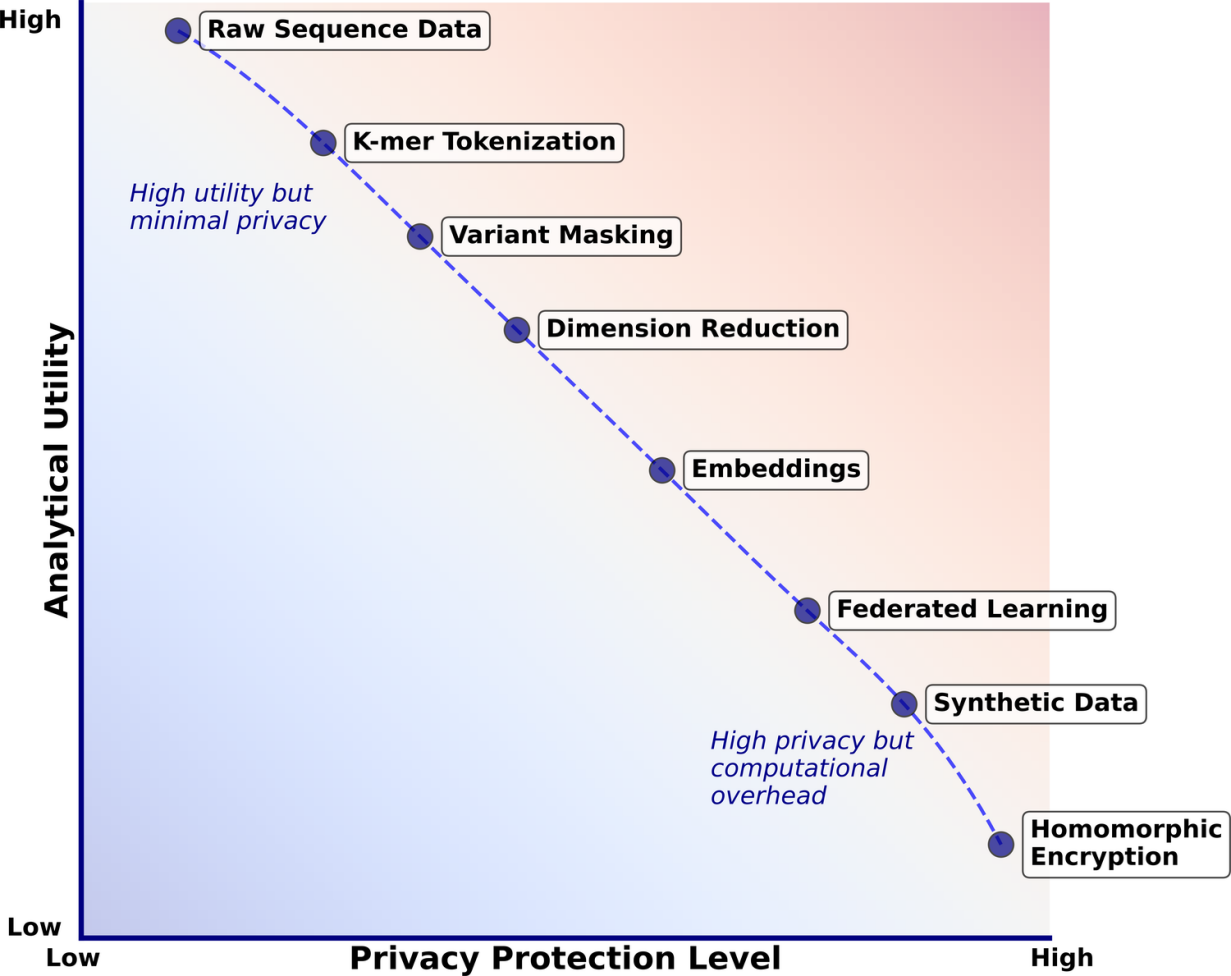
There is a spectrum of privacy-preserving techniques for GLMs that provide different levels of privacy protection and analytical utility. Techniques range from handling raw sequence data (high utility, since it can be processed in any way, but minimal privacy) to homomorphic encryption (high privacy but high overhead to extract meaning)

Figure 4 illustrates the spectrum of privacy-preserving techniques for GLMs, highlighting the inherent trade-off between privacy protection and analytical utility. As the methodology progress from simply exposing raw sequence data as a baseline toward to sophisticated methods like homomorphic encryption (Sarkar et al. 2023), they provide increasing privacy safeguards but often at the cost of reduced utility or increased computational complexity.

Sitting in between scoring methods and encryption, embeddings and transformations are other powerful methods for abstracting genomic data in ways that enhance both usability and privacy. By converting high-dimensional, sensitive genomic sequences into lower-dimensional vector representations—known as embeddings—researchers can retain essential biological information while obscuring personally identifiable details (Sultan et al. 2022). Embeddings function as a form of dimensionality reduction that captures the semantic relationships between genomic elements while creating a layer of abstraction that helps protect individual identity. LLM choice should be carefully considered since some techniques could specifically embed the SNP type and thus be identifiable (Cahyawijaya et al. 2022).

Embeddings can be designed to select relevant genomic features (e.g., variant patterns associated with phenotypes, structural motifs, or evolutionary signals) for downstream tasks like phenotype prediction (e.g. disease) (van Hilten et al. 2021; Benegas et al. 2024) or gene clustering (Refahi et al. 2025), without revealing the raw DNA sequences. This selective feature representation allows researchers to focus on biologically relevant patterns while minimizing exposure of sensitive personal genetic information, creating a balance between analytical utility and privacy protection. Embeddings can be learned through unsupervised methods such as autoencoders or sequence-based neural models (e.g., transformer architectures trained on genomic data), or through supervised models trained to optimize task-specific objectives like disease classification. These diverse learning approaches offer flexibility in how genomic data is represented, allowing researchers to tailor the embedding method to the specific privacy requirements and analytical goals of their studies. However, one must be aware that in any study with heterogeneous samples of data, it may be possible to use statistical techniques and rarely occurring alleles to trace back to individual identification (Lippert et al. 2017) and perhaps geographic location (Battey et al. 2020).

#### Developing model interpretability methods adapted to GLMs

There are many ways to explain GLMs so that they can provide reasoning for their decisions, such as attention mechanisms, attribution techniques, sequence feature extraction, and perturbation and ablation studies (on the sequence or higher level features). These explainability approaches serve a critical function in genomics by connecting the statistical patterns learned by models to biological mechanisms that scientists can validate experimentally. One approach is to extend transformer visualizers, where the intermediate output of the core (Ji et al. 2021, Zvyagin et al. 2022, Zhao et al. 2021, Yang et al. 2022) attention layers of GLMs to visualized to show which parts of a sequence the model “attends” to when making a prediction. Examples of transformer visualizers applied to GLMs include DNABERT (Ji et al. 2021), GenSLMs (Zvyagin et al. 2022, Zhao et al. 2021), and LOGO (Yang et al. 2022). If a model predicts that a region is a promoter, for example, attention heads might focus on TATA boxes or CpG islands. Attention visualizations should be interpreted with caution: while they can provide an intuitive representation of model behavior, they may not perfectly align with the model’s actual decision-making process or underlying biological reality.

There have also been important advances in creating explainability methods that can work with the complex, nonlinear AI models, including LLMs and GLMs. Studies have compared various attribution methods such as DeepLIFT, Integrated Gradients, and GradCAM—techniques that quantify the contribution of each input feature to the model’s output by tracking gradients or activation differences through the model’s neural network layers (Prakash et al. 2021; Koo and Ploenzke 2021). SQUID introduces a novel approach using surrogate models that approximate neural network functions to improve attribution accuracy (Seitz et al. 2024). Computational methods like Saliency Maps (Simonyan et al. 2014), LIME (Local Interpretable Model-agnostic Explanations) (Ribeiro et al. 2016; Kabir et al. 2024), and SHAP (SHapley Additive explanations) (Johnsen et al. 2023) can show how each nucleotide, *k*-mer, or sequence region contributes to a prediction. If one has access to the original sequences that contribute to a clustered or classification group of embeddings, it could be possible to find and extract what SNPs, or other motifs, that this group has in common. Finally, perturbation and ablation studies can identify relevant features in a brute force manner; these include, e.g., sequence masking/randomization (Linder et al. 2022), in silico mutagenesis (Demajo et al. 2024), and attention-head/neuron/task-specific ablation (Pochinkov et al. 2024). These approaches may offer a more direct test of causality than correlation-based methods, helping researchers distinguish between predictive and mechanistically important genomic features.

As explained above, *post-hoc* methods must be carefully applied to avoid unreliability or merely creating the appearance of understanding. The development of explanation techniques should therefore include (1) comparative validation across multiple methods to establish consistency and reliability, as no single one provides complete insight; (2) integration of biological prior knowledge to constrain explanations within mechanistically plausible frameworks; and (3) development of benchmarks with ground truth explanations tailored to genomic contexts to quantitatively evaluate explanation fidelity. Successful approaches will likely combine computational explainability with experimental validation methods, such as using deep mutational scanning (Maes et al. 2023) and targeted mutagenesis in model organisms (Maes et al. 2023), to verify that model-identified features causally impact biological function rather than representing statistical artifacts.

## Conclusions

GLMs represent a powerful convergence of AI and genomics with transformative potential for biomedicine and biotechnology. However, as discussed in this paper, GLMs create unique regulatory challenges that neither AI governance nor traditional genomic privacy frameworks fully address. The tensions between these domains—from privacy and consent to bias, interpretability, and liability—require innovative solutions that bridge these regulatory gaps.

We propose a layered governance approach that combines technical safeguards with policy innovations tailored to the unique characteristics of GLMs. As summarized in Table 2, these solutions directly address the key governance tensions posed by GLMs: Privacy-preserving data representations tackle the inference capabilities that undermine anonymization; regulatory sandboxes provide controlled environments to explore consent models for generative insights. Interpretability techniques address the black-box nature of GLMs that undermines clinical trust. And certification frameworks clarify liability across developers, providers, and institutions.

Layered governance will only be effective if it can balance enabling beneficial innovation with protecting individuals from potential harms related to privacy breaches, discrimination, and biosecurity concerns. As GLMs evolve and their applications expand beyond genomics into protein modeling and synthetic biology (Nguyen et al. 2024), proactive governance becomes increasingly urgent. By fostering collaboration between AI experts, genomics researchers, ethicists, clinicians, and policymakers, research communities, non-profit organizations, and government agencies can develop adaptive frameworks that address GLMs' distinct challenges. Ultimately, maximizing the benefits of GLMs without imposing unacceptable social and individual costs will depend not merely on technical advances. It will also require the collective ability to create and adopt governance structures that are as sophisticated and forward-thinking as the technologies themselves.

### Technical terms

*Attention mechanism*—A component of neural networks that allows models to focus on specific parts of input data when making predictions.

*Black box model*—A machine learning model whose internal decision-making process is not easily interpretable or explainable.

*Byte-pair encoding*—A data compression technique used in natural language processing to create variable-length tokens.

*Direct-to-consumer genetic testing*—Commercial genetic testing services that provide genetic information directly to consumers without requiring healthcare provider involvement.

*Embeddings*—Lower-dimensional vector representations of high-dimensional data that preserve important relationships while reducing complexity.

*Federated learning*—A distributed machine learning approach where multiple parties train a shared model on their local data without sharing the raw data itself.; only model parameters and updates are exchanged across the network.

*Homomorphic encryption*—A form of encryption that allows computations to be performed on encrypted data without decrypting it first.

*k-mer*—A subsequence of length *k* extracted from a longer DNA sequence.

*Masked language modeling*—A training technique where random tokens in a sequence are hidden and the model learns to predict them from context.

*Post-hoc explanation*—Explanations generated after a model has been trained to interpret its decisions, rather than being built into the model architecture.

*Polygenic risk score*—A numerical estimate of an individual's genetic predisposition to a trait or disease based on multiple genetic variants.

*Pretraining*—The initial phase of training a language model on large datasets before fine-tuning for specific tasks.

*Regulatory sandbox*—A controlled testing environment where innovative technologies can be developed with regulatory flexibility under supervision.

*Soft governance*—Non-binding regulatory approaches including ethical frameworks, guidelines, and industry standards.

*Tokenization*—The process of breaking down sequences (text or DNA) into smaller units (called “tokens”) for processing by language models.

*Transformer architecture*—A neural network architecture that uses self-attention mechanisms to process sequential data.

## Data Availability

No datasets were generated or analysed during the current study.

## Abbreviations

ACMG: American College of Medical Genetics and Genomics
AI: Artificial intelligence
AIA: Algorithmic impact assessment
ARCCH: AI and Regulation Common Capacity Hub
BERT: Bidirectional encoder representations from transformers
D2C: Direct-to-consumer (genetic testing)
EU: European Union
FDA: Food and Drug Administration (United States)
GA4GH: Global Alliance for Genomics and Health
GDPR: General Data Protection Regulation (European Union)
GINA: Genetic Information Nondiscrimination Act (United States)
GLM: Genome language model
GPT: Generative pre-trained transformer
HIPAA: Health Insurance Portability and Accountability Act (United States)
ICGC: International Cancer Genome Consortium
LIME: Local interpretable model-agnostic explanations
LLM: Large language model
NIST: National Institute of Standards and Technology (United States)
OECD: Organisation for Economic Co-operation and Development
PII: Personally identifiable information
SHAP: SHapley Additive exPlanations
SNP: Single nucleotide polymorphism
UNESCO: United Nations Educational, Scientific and Cultural Organization
WHO: World Health Organization
